# 

**Published:** 1860-10

**Authors:** 


New Series, Vol. 3, No. 10. Whole Series, Vol. 17, N[illegible]

### THE CHICAGO

### MEDICAL JOURNA[illegible]

DANIEL BRAINARD, M. D.,
EPHRAIM INGALS, M. D.,
EDITORS AND PROPRIETORS.

OCTOBER, 1860.

CHICAGO:
J. W. HYATT, Jr., BOOK AND JOB PRINTER, 23 LAKE STREE[illegible]
To whom all advertisements for insertion in the Journal may be addressed.

PUBLISHED MONTHLY, AT TWO DOLLARS PER ANNUM, IN ADVA[illegible]

